# Estimation of a significance threshold for genome-wide association studies

**DOI:** 10.1186/s12864-019-5992-7

**Published:** 2019-07-29

**Authors:** Avjinder S. Kaler, Larry C. Purcell

**Affiliations:** Department of Crop, Soil, and Environmental Sciences, University of Arkansas, Fayetteville, AR 72704 USA

**Keywords:** Genome-wide association studies, Significant threshold, Bonferroni correction, False discovery rate, Heritability, Single nucleotide polymorphisms

## Abstract

**Background:**

Selection of an appropriate statistical significance threshold in genome-wide association studies is critical to differentiate true positives from false positives and false negatives. Different multiple testing comparison methods have been developed to determine the significance threshold; however, these methods may be overly conservative and may lead to an increase in false negatives. Here, we developed an empirical formula to determine the statistical significance threshold that is based on the marker-based heritability of the trait. To develop a formula for a significance threshold, we used 45 simulated traits in soybean, maize, and rice that varied in both broad sense heritability and the number of QTLs.

**Results:**

A formula to determine a significance threshold was developed based on a regression equation that used one independent variable, marker-based heritability, and one response variable, − log_10_ (*P*)-values. For all species, the threshold –log_10_ (*P*)-values increased as both marker-based and broad-sense heritability increased. Higher broad sense heritability in these crops resulted in higher significant threshold values. Among crop species, maize, with a lower linkage disequilibrium pattern, had higher significant threshold values as compared to soybean and rice.

**Conclusions:**

Our formula was less conservative and identified more true positive associations than the false discovery rate and Bonferroni correction methods.

**Electronic supplementary material:**

The online version of this article (10.1186/s12864-019-5992-7) contains supplementary material, which is available to authorized users.

## Background

Linkage mapping (LM) and genome-wide association studies (GWAS) are the two most popular methods to decipher genetic architectures of complex traits in crops [1]. With advancements in high throughput genotyping and sequencing technologies, single nucleotide polymorphisms (SNPs) provide relatively low cost and dense marker coverage across various genomes [2]. Association mapping has several advantages over the traditional LM, including increased mapping resolution, broader allele coverage, and reduced time and costs to establish tedious and expensive biparental mapping populations [3].

A major problem in GWAS is false positives that arise from population structure and family relatedness. Several statistical models have been developed to control false positives in GWAS. Mixed linear model (MLM) has become the most popular approach with the ability to consider population structure and family relatedness [3, 4]. Since the publication of MLM for GWAS [3], many MLM-based methods have been developed. All these methods are single-locus, which test one marker at a time, and these methods fail to match the true genetic model of complex traits that are controlled by many loci simultaneously. To overcome this problem, multi-locus models, including FASTmrEMMAa [5], ISIS EM-BLASSO [6], pLARmEB [7], pKWmEB [8], LASSO [9], and FarmCPU [10], have been developed.

Determining the correct *P*-value threshold for statistical significance is critical to differentiate true positives from false positives and false negatives. To determine the statistical significance threshold in GWAS, different statistical procedures accounting for multiple testing have been proposed, including the Bonferroni correction, Sidak correction, False Discovery Rate (FDR), permutation test, and Bayesian approaches. Bonferroni correction and FDR [11–15] are the two most commonly used methods for crops. All of these methods limit type 1 errors (false-positives), but they almost certainly inflate type 2 errors (false negatives) [16].

The Bonferroni correction method is considered the most conservative method for selecting a threshold *P*-value due to the assumption that every genetic variant tested is independent of the rest. The False Discovery Rate controls the expected proportion of false positives among the rejected null hypotheses and is a popular, less conservative approach compared to the Bonferroni correction [15]. However, FDR also assumes independence of hypotheses; therefore, if many SNPs in strong linkage disequilibrium (LD) are present on an array, it can suffer from a loss of statistical power and generate false negatives [17]. An imbalance of error rates permitting an excess of false negatives may be more problematic in the long term because type 1 errors are more easily identified in subsequent studies, and the resources necessary to perform other large GWAS needed to overcome the bias toward type 2 errors are finite [16]. Additionally, the variants tested in a study are inevitably dependent on population-specific factors, such as LD pattern and minor allele frequency (MAF), suggesting that the appropriate threshold for genome-wide significance might vary for different populations and crop species. For example, the threshold for a crop with a lower LD pattern, such as maize (*Zea mays* L.), should be more stringent than a population with higher LD pattern, such as soybean (*Glycine max* L.) or rice (*Oryza sativa* L.), as the number of independent markers tends to be greater in maize than soybean. The LD decay rate (*r*^2^ = 0.25 level) was much greater in maize (1 kb) [18] than soybean (150 kb in euchromatic and 5,000 kb heterochromatic regions) [19–21]. or rice (123 kb) [22]. Therefore, there is a need to develop a method that can select an appropriate significant threshold value for GWAS to differentiate true positives from false positives and false negatives.

As trait complexity increases, the number of loci affecting the trait increases along with environmental interactions with an expected decrease in heritability. Conversely, for less complex traits, fewer loci affect the trait, there is less interaction with the environment, and there is an expected increase in heritability. For a trait with a high heritability, the threshold value for significance of associating loci with a trait would have high – log_10_ (*P*)-values, and vice versa for a complex trait with low heritability.

Here, we develop an empirical formula to determine the statistical significance thresholds that is based on the marker-based heritability of the trait. The objective of this study was to develop an empirical formula that can determine the statistical significance thresholds for GWAS using a large number of simulated phenotypes that varied in heritability and the number of QTLs for soybean, maize, and rice. These crops were selected because of differences in LD pattern with maize having a lower LD pattern compared with soybean and rice. The phenotypes were simulated and associated with freely-available SNP marker datasets for all these crops.

## Results and discussion

In this study, we developed a method to determine the significant threshold value for GWAS using the 45 simulated phenotypic traits that varied in both the broad sense heritability and the number of QTLs in three crop species that differed in their LD patterns. We repeated the simulation of these traits 10 times so that simulated QTLs were randomly assigned to different parts of the genome in order to obtain unbiased results.

**Fig. 1:**
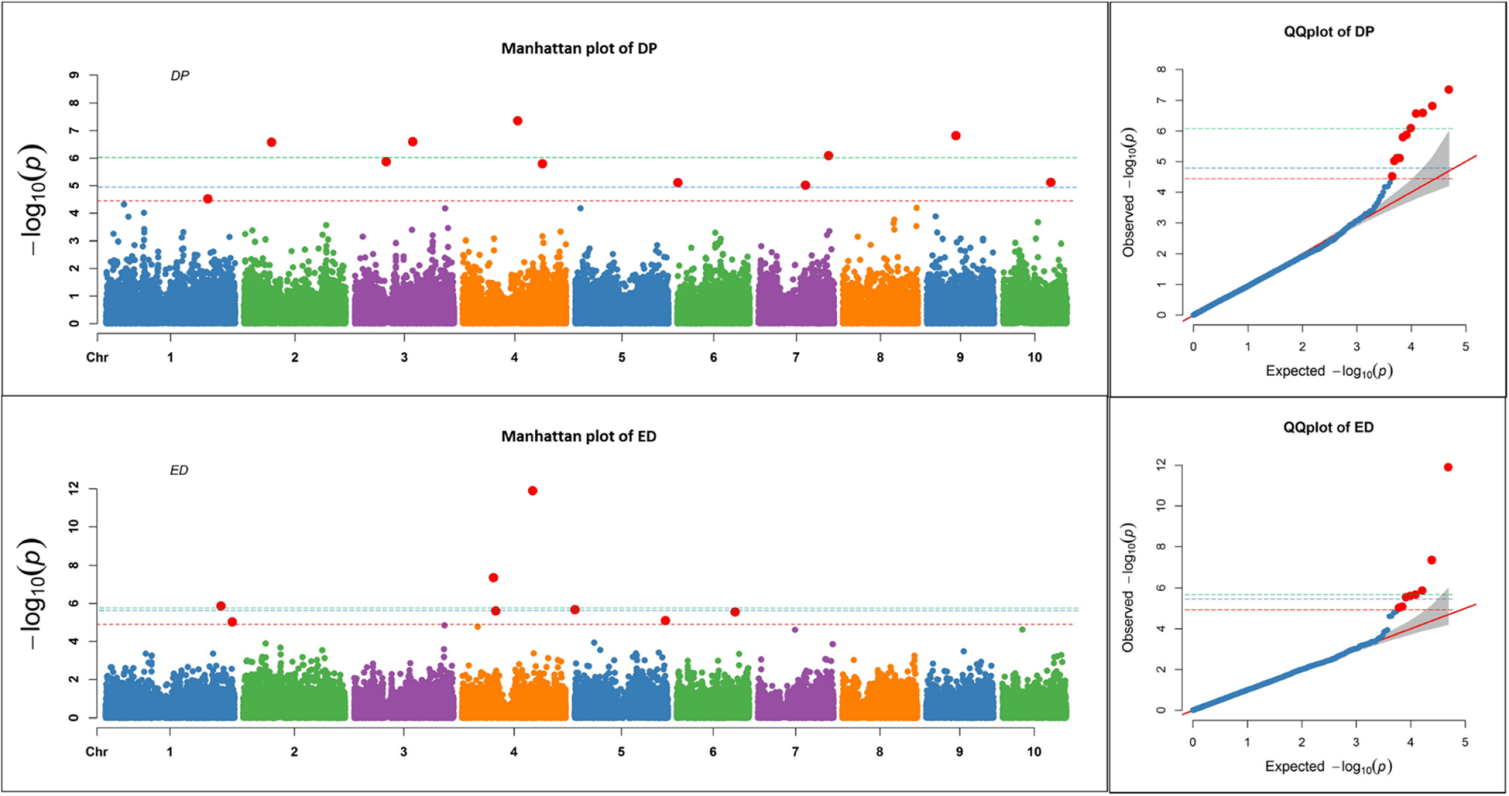
Manhattan plots of -Log10 (*P*) vs. chromosomal position of SNP markers associated with ear diameter (ED) and days to pollination (DP), and quantile-quantile (QQ) plots in maize from the Fixed and random model Circulating Probability Unification (FarmCPU). Marker-based heritability was 66.8% for DP and 84.9% for ED. A red line represents the significant threshold (−Log10 (*P*) values: 4.89 for DP and 5.49 for ED), which was determined using our formula based on the marker-based heritability, a blue line represents the threshold from the FDR, and a green line represents the threshold from the Bonferroni correction method

For the same simulated trait in different repetitions, there were different marker-based heritabilities and different significant – log_10_ (*P*)-values (where all simulated QTLs in that trait were present) (Fig. 1). There were strong positive associations between broad sense heritability and significant threshold values. That is, the higher the broad sense heritability, the higher the – log_10_ (*P*)-values for all three crops (Table 1). Significant threshold values (−log_10_ (*P*)) also increased among the crop species for these simulated traits as the LD decreased. Specifically, maize had higher significant threshold (−log_10_ (*P*)) values as compared to soybean and rice for simulated traits when they had more than 50% broad sense heritability (Table 1), which corresponded inversely with LD patterns.

**Table 1:** Significant *P*-values (−Log_10_ *P*-value) from FarmCPU where all 10 associated QTLs with 9 simulated traits varied in broad sense heritability (H = 10, 20, 30, 40, 50, 60, 70, 80, 90%) in maize, soybean, and rice

	Maize	Rice	Soybean
Simulated Traits			
H10_Q10	3.54	3.94	3.17
H20_Q10	3.67	3.91	3.58
H30_Q10	4.00	4.05	3.64
H40_Q10	4.17	4.23	3.84
H50_Q10	4.68	4.29	4.03
H60_Q10	4.84	4.45	4.12
H70_Q10	5.07	4.65	4.73
H80_Q10	7.02	5.39	5.62
H90_Q10	15.08	7.45	7.95

Using both broad-sense heritability and marker-based heritability as independent variables and the selected significant threshold (−log_10_ (*P*)) value as the response variable in the multiple regression analysis, we obtained an equation for determining significant threshold values in GWAS for each crop. We observed that marker-based heritability showed a significant effect on the response variable (*P* < 0.05) (Table 2), but there was no significant effect of broad-sense heritability. Therefore, only marker-based heritability was included in the regression eq. (Y = a + bX), where Y was the significant threshold (−log_10_ *P*-value), a was the intercept, and b was the slope of the regression coefficient for the marker-based heritability (X) in maize, soybean, and rice. Table 2 shows the intercept and slope of regression equations in 10 out of 100 different repetitions. We used the raw value of the intercept and slope from 100 different repetitions to develop the final formula. Although, the fit for regression equation was poor for maize (R^2^ = 0.14) and rice (R^2^ = 0.16), and was moderate for soybean (R^2^ = 0.35), these regressions were highly significant (*P* < 0.0001) and indicate that the predictor variables still provide information about the response even though data points fall further from the regression line.

**Table 2:** Intercept (a) and slope (b) values of regression eqs. (Y = a + bX), predicting the significant threshold (−Log_10_ *P*-value), as a function of the marker-based heritability (X) in maize, soybean, and rice

	Maize				Soybean				Rice			
Repetition	Constant	Slope	*R*^2^	*P*-value	Constant	Slope	*R*^2^	*P*-value	Constant	Slope	*R*^2^	*P*-value
1	2.49	0.032	0.15	0.008	2.10	0.027	0.45	4.4e-07	2.59	0.016	0.11	0.02
2	2.91	0.022	0.10	0.03	2.05	0.030	0.40	2.9e-06	2.58	0.015	0.11	0.02
3	2.71	0.031	0.14	0.01	2.09	0.033	0.22	0.001	2.52	0.017	0.18	0.004
4	2.93	0.019	0.13	0.01	2.20	0.026	0.36	1.3e-05	2.26	0.021	0.19	0.003
5	2.75	0.024	0.11	0.02	2.01	0.032	0.40	3.7e-06	2.33	0.022	0.20	0.002
6	2.88	0.022	0.09	0.04	2.28	0.027	0.42	1.3e-06	2.62	0.016	0.13	0.01
7	2.87	0.022	0.15	0.008	2.18	0.026	0.40	3.6e-06	2.62	0.020	0.11	0.02
8	2.75	0.026	0.13	0.01	2.16	0.027	0.36	1.5e-05	2.41	0.017	0.21	0.001
9	2.47	0.034	0.12	0.01	2.10	0.030	0.39	3.9e-06	2.64	0.017	0.14	0.01
10	2.68	0.027	0.13	0.01	2.14	0.028	0.39	4.0e-06	2.51	0.018	0.19	0.003
All Raw Data	2.77	0.025	0.14	7.6e-15	2.16	0.028	0.35	< 2.2e-16	2.53	0.017	0.15	2.8e-16

**Fig. 2:**
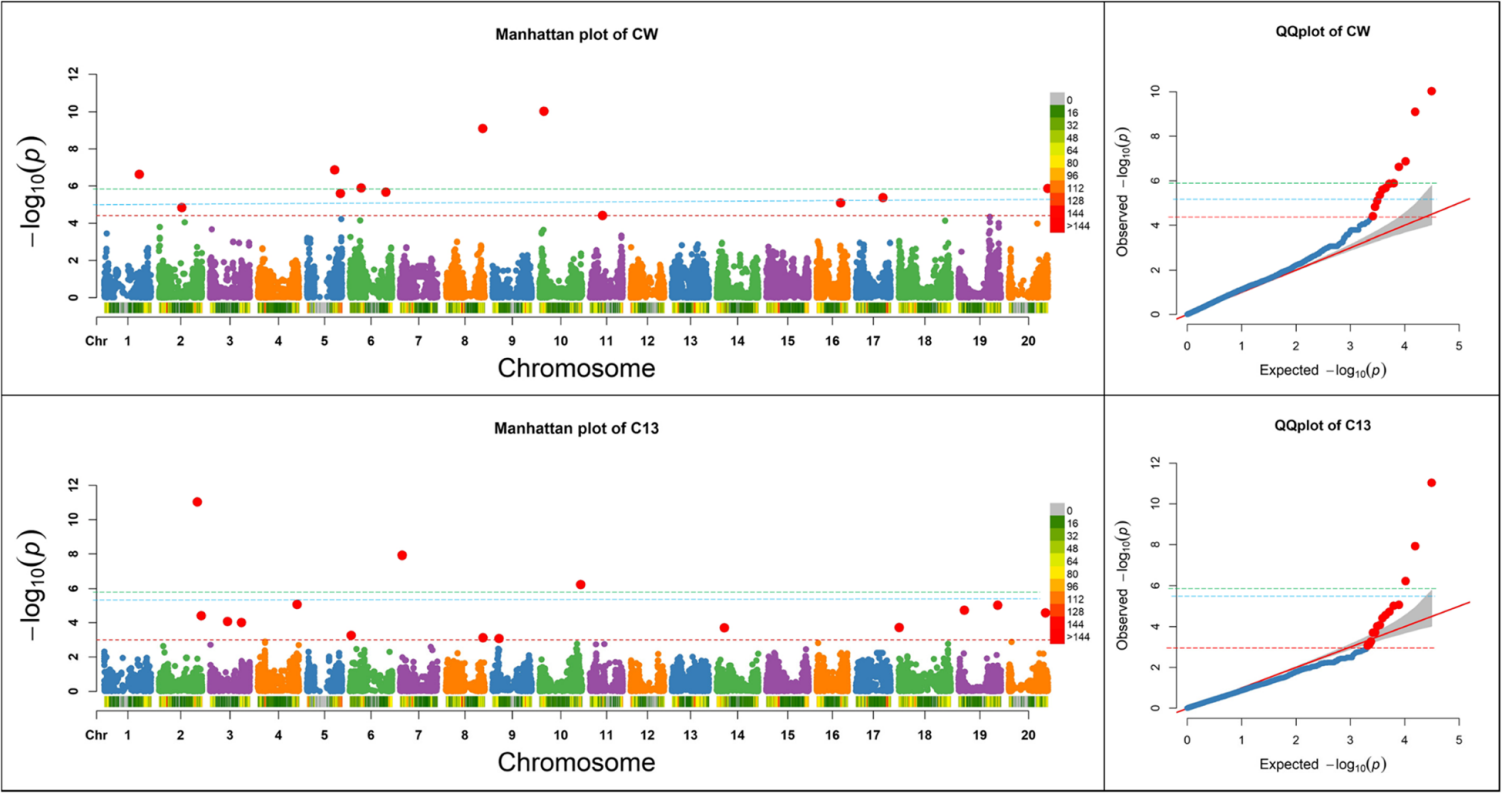
Manhattan plots of -Log10 (*P*) vs. chromosomal position of SNP markers associated with canopy wilting (CW) and carbon isotope ratio (C13), and quantile-quantile (QQ) plots in soybean from the Fixed and random model Circulating Probability Unification (FarmCPU). Marker-based heritability was 28.6% for C13 and 77.8% for CW. A red line represents the significant threshold (−Log10 (*P*) values: 2.96 for C13 and 4.39 for CW), which was determined using our formula based on the marker-based heritability, a blue line represents the threshold from the FDR, and a green line represents the threshold from the Bonferroni correction method

**Fig. 3:**
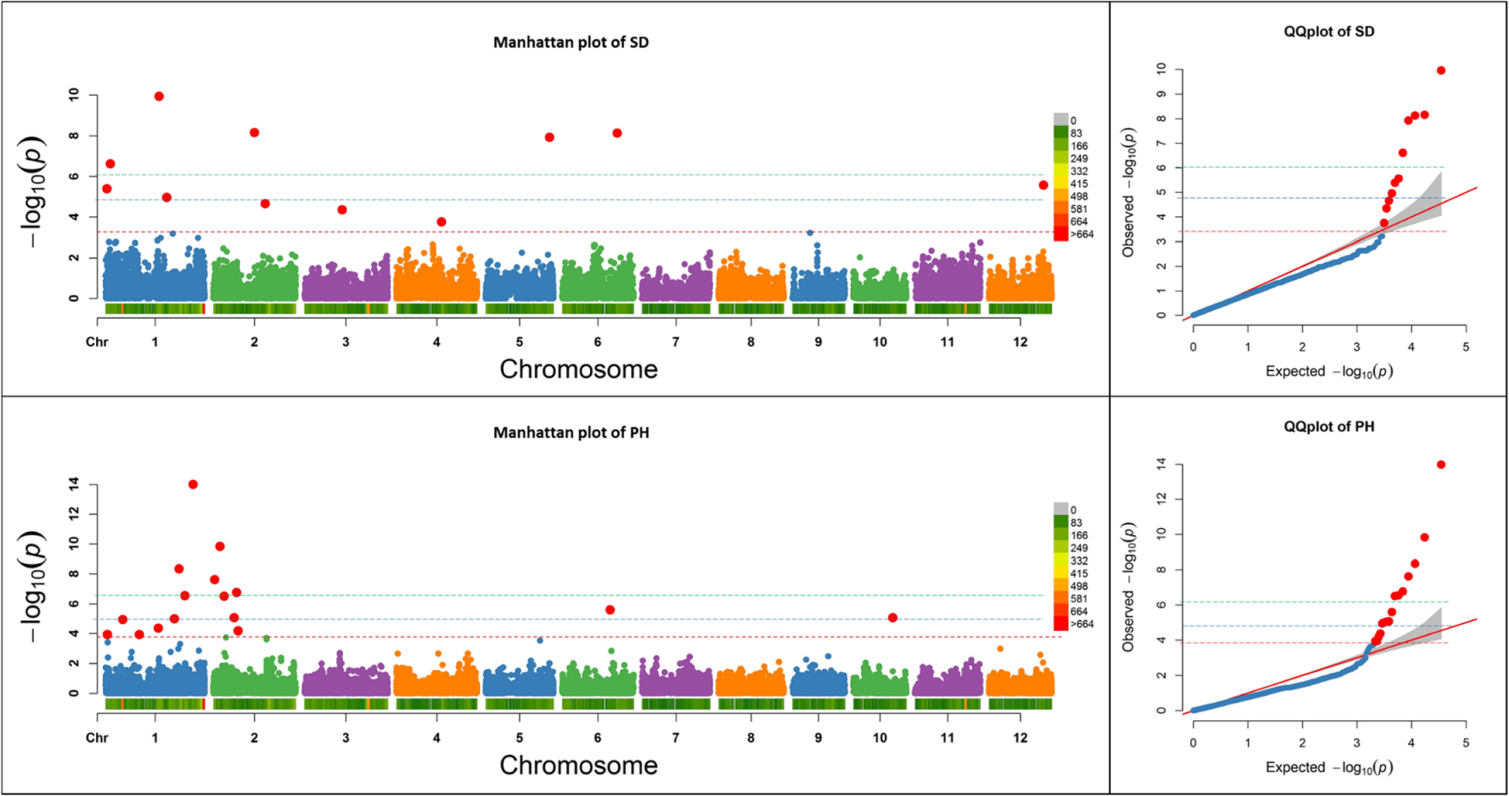
Manhattan plots of -Log10 (*P*) vs. chromosomal position of SNP markers associated with seeds per panicle (SD) and plant height (PH), and quantile-quantile (QQ) plots in soybean from the Fixed and random model Circulating Probability Unification (FarmCPU). Marker-based heritability was 42.8% for SD and 68.8% for PH. A red line represents the significant threshold (−Log10 (*P*) values: 3.28 for SD and 3.75 for PH), which was determined using our formula based on the marker-based heritability, a blue line represents the threshold from the FDR, and a green line represents the threshold from the Bonferroni correction method

For datasets based on previously reported results, estimated marker-based heritability was 66.8% for DP and 84.9% for ED in maize, 28.6% for C13 and 77.8% for CW in soybean, and 42.8% for SD and 68.8% for PH in rice. These marker-based heritability values were used to determine significant threshold (−log_10_ (*P*)) values as shown in Figs. 1, 2, and 3 based upon the regression equation for each respective crop in Table 2. Additional file 1: Figure S1 shows the relationship between response significant threshold and marker-based heritability in maize, soybean, and rice.

Manhattan and QQ plots in Figs. 1–3 show the comparisons of our formula based threshold (a red line) with FDR (a blue line) and Bonferroni correction (a green line) methods using previously published datasets for DP and ED in maize (Fig. 1), C13, and CW in soybean (Fig. 2), and SD and PH in rice (Fig. 3). The sharp break upwards in QQ plots indicates where the *P*-value threshold for true associations begin [19]. The *P*-value threshold determined using our method captured more true positives than the FDR and Bonferroni corrections methods as indicated by being closer to the breakpoint at which the observed *P*-value increases sharply. Some of the extra markers that were identified for previously published datasets by our formula-based threshold, were coincident in the same genomic region of previously reported QTLs studies for that trait (data not shown). Higher broad sense heritability traits in these crops had higher significant threshold values. Among crop species, maize, with a lower LD pattern, had higher significant threshold values as compared to soybean and rice (Figs. 1, 2, 3).

**Fig. 4:**
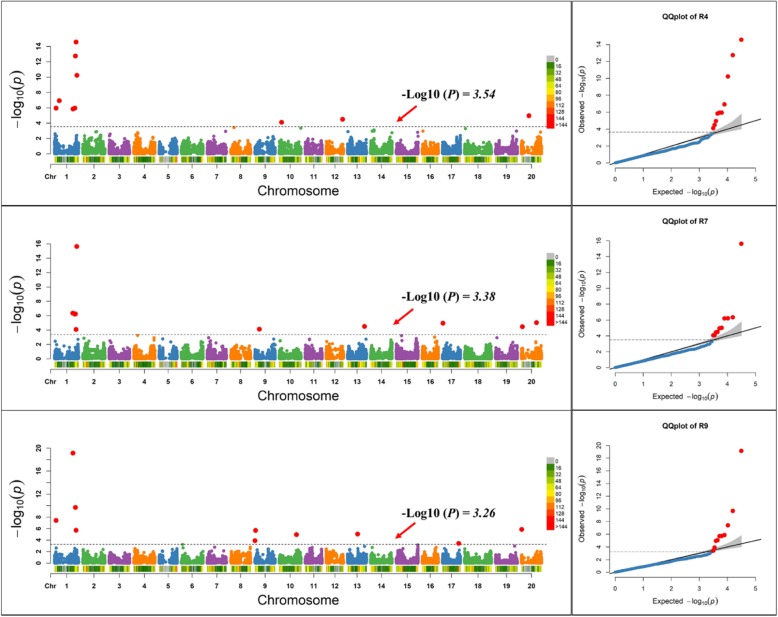
Manhattan plots of -Log10 (*P*) vs. chromosomal position of SNP markers associated with soybean simulated trait that had 60% heritability and 10 QTLs from three randomly selected repetitions (R4, R7, and R9) using the real SNP markers dataset, and quantile-quantile (QQ) plots in soybean from the Fixed and random model Circulating Probability Unification (FarmCPU). Estimated marker-based heritability of this simulated trait was 48.6% in R4, 43.2% in R7, and 39.1% in R9, which was used in the formula to select significant thresholds -Log10 (*P*) values, such 3.54 in R4, 3.38 in R7, and 3.26 in R9. A red line represents the significant threshold values in these different repetitions. For all three repetitions, 10 markers were identified above the threshold value but in some cases these may be hidden behind other markers

We also used the one simulated trait in soybean that had 60% broad sense heritability and 10 QTLs in three randomly selected repetitions (R4, R7, and R9) to determine if our formula accurately estimated threshold *P*-values identified in the 10 simulated QTLs. A simulated trait in different repetitions had different marker-based heritability values of 48.6% (R4), 43.2% (R7), and 39.1% (R9). Using this marker-based heritability, significant threshold *P*-values were determined for the simulated trait in all three repetitions. Results indicated that our formula-based threshold values identified 10 QTLs for this simulated trait in these three repetitions across different parts of the genome (Fig. 4). The sharp break upwards in QQ plots from this simulated trait in all three repetitions also indicated that our formula-based threshold values identified 10 true associations (Fig. 4).

Using the equation developed from marker-based heritability, we evaluated our threshold *P*-values with other multiple testing comparison methods using the GWAS results from the previously-published phenotypic datasets in maize [23], soybean [19, 20], and rice [24]. The results indicated that selection of significant threshold values based on our formula were less conservative than other multiple comparisons in controlling both false positives and false negatives (Table 3). Table 3 shows the comparisons of having no correction (uncorrected *P* ≤ 0.05) with our formula, Bonferroni correction, and FDR. Because Bonferroni, Šidák, Hommel, and Hochberg corrections had similar results, and False Discovery Rate and Positive False Discovery Rate had similar results, only Bonferroni correction and FDR are shown in Table 3. For all traits in maize, soybean, and rice, our formula was less conservative in identifying true positive associations as compared to both FDR and Bonferroni correction methods (Table 3). The column marked none in Table 3 represents the selection of significant SNPs at a threshold value (−log_10_ *P* ≥ 3.5), which was the arbitrary selection. Our formula identified a greater number of markers than the uncorrected method for the C13 trait in soybean, which might be due to the generation of false negatives in the uncorrected method.

**Table 3:** Comparisons of the number of markers identified as significant based upon various criteria

Crop	Trait	None	MBH	Bon	FDR
Maize	DP	24	11	5	10
	ED	19	8	5	6
Soybean	C13	12	15	3	3
	CW	38	13	6	11
Rice	SD	11	11	5	8
	PH	21	17	7	12

The column marked ‘None’ represents the selection of significant SNPs at an arbitrary threshold value (−Log_10_ *P* ≥ 3.5). The column marked MBH represents the number of markers identified using the marker-based-heritability-regression method. Columns marked Bon and FDR refer to Bonferroni corrections and positive False Discovery Rate, respectively, for the number of significant markers that were selected based on a cutoff of 0.05. Data sets for these analysis were previously published reports for days to pollinations (DP) and ear diameter (ED) in maize, carbon isotope ratio (C13) and canopy wilting (CW) in soybean, and seeds per panicle (SD) and plant height (PH) in rice

These results indicate that selection of significant threshold values vary in different populations and crop species, which depend on the heritability of the trait in a particular environment. The GWAS results for these comparisons were obtained from the FarmCPU model because this multi-locus model effectively controlled false positives that arise from population structure and family relatedness as compared to all MLM models (Kaler et al. unpublished results), which are single-locus models.

## Conclusions

We developed a simple method for determining the threshold *P*-value for GWAS based upon the marker-based heritability of a trait in a specific environment. This method is simple and robust across a wide range of heritabilities and species with different LD. This method is less conservative and captures more true positives as compared to more conservative methods such as FDR and Bonferroni corrections.

## Methods

### Data collection

To develop a formula for a significance threshold, we used 45 simulated traits in soybean, maize, and rice that varied in broad sense heritability and the number of QTLs (Q). We used an R code script for simulation, where real genotypic data of each crop was used and different number of QTLs and heritability were assigned to create a simulated phenotype. In soybean, genotypic data consisted of 42,509 SNP markers (www.soybase.org) for 346 accessions that were previously reported by Kaler et al. [19, 20]. Phenotypic data for canopy wilting and carbon isotope ratio for these 346 accessions is provided in Additional file 1: Table S1. In maize, genotypic data consisted of 50,896 SNP markers for 273 accessions [25]. In rice, genotypic data consisted of 44,100 SNP markers for 352 accessions that were obtained from two projects: (1) *Oryza*SNP project, an oligomer array-based re-sequencing effort using Perlegen Sciences technology, and (2) BAC clone Sanger sequencing of wild species from the OMAP project [24].

The 45 phenotypic traits were simulated using a R-code script (Additional file 1: Table S2). The simulations represent nine different combinations of broad sense heritability (10, 20, 30, 40, 50, 60, 70, 80, and 90%), and five different combinations of the number of QTLs associated with the simulated trait (10, 20, 30, 40, and 50 QTLs). These 45 simulations were repeated 100 times each.

### Formula development

A formula to determine a significance threshold was developed based on a multiple regression equation that used two independent variables, broad-sense heritability and marker-based heritability, and one response variable, − log_10_ (*P*)-values. Broad-sense heritability was the heritability that was used to simulate the trait, and marker-based heritability was estimated using genetic variance determined from a simulated trait and genotypic marker data [26] that were obtained from the GAPIT R package [27]. In the GAPIT package, the MLM model can be described as follows: *Y* = *Xβ* + *Zu* + *e*, where where Y is the vector of observed phenotypes; β is an unknown vector containing fixed effects, including the genetic marker, population structure (Q), and the intercept; u is an unknown vector of random additive genetic effects from multiple background QTL for individuals/lines; X and Z are the known design matrices; and e is the unobserved vector of residuals. The u and e vectors are assumed to be normally distributed with a null mean and a variance of: $$ Var\ \left(\begin{array}{c}u\\ {}e\end{array}\right)=\left(\begin{array}{cc}G& 0\\ {}0& R\end{array}\right) $$, where G = σ^2^_a_K with σ^2^_a_ as the additive genetic variance and K as the kinship matrix. Homogeneous variance is assumed for the residual effect; i.e., R = σ^2^_e_I, where σ^2^_e_ is the residual variance. The proportion of the total variance explained by the genetic variance is defined as marker-based heritability.

The response variable was the – log_10_ (*P*)-value determined from the association analysis of a simulated trait that identified the number of QTLs for that simulated trait. For example, if a simulated trait had 10 QTLs, then the significant – log_10_ (*P*)-value was selected that identified these 10 QTLs after performing association analysis using the FarmCPU model [10]. The FarmCPU is a multi-locus model that was used for association analysis because it performs better than other models in controlling false positives and false negatives [19].

### Validation and comparison of the formula

We validated this formula using the GWAS results from previously-published phenotypic datasets in soybean, maize, and rice. The GWAS results were obtained after performing association analysis on the datasets including carbon isotope ratio (C13) [20] and canopy wilting (CW) [19] in soybean, days to pollination (DP) and ear diameter (ED) in maize [23], and seeds per panicle (SD) and plant height (PH) in rice [24]. We also compared our formula with different multiple testing comparisons, including Bonferroni, Šidák, Hommel, Hochberg, False Discovery Rate, and Positive False Discovery Rate [11–15] with a significant cut off of 0.05. The GWAS results obtained from compressed mixed linear model (CMLM) and FarmCPU models were also used in these comparisons.

## Additional file

Additional file 1: **Figure S1.** Scatter plots between significant threshold and marker-based heritability in maize, soybean, and rice. **Table S1.** Phenotypic data of canopy wilting (CW) and carbon isotope ratio (C13) from 346 soybean accessions previously reported by Kaler et al. (19, 20). **Table S2.** The R code script used for trait simulation for rice data. Similar programming can be used for other crops by changing the genotypic data. (DOCX 176 kb)

## Data Availability

The R code script used for trait simulation in this study is provided using as an example the script for rice data. Similar programming can be used for other crops by changing the genotypic data. The 346 soybean genotypes used in this study are part of 19,652 *G. max* and *G. soja* accessions genotyped with SoySNP50K iSelect Beadchip (http://www.soybase.org/snps/download.php). Additional file 1: Table S1 provides phenotype data for soybean canopy wilting and carbon isotope ratio. Similarly, the 279 maize genotypes and 352 rice genotypes are also available to the public at the website, https://www.panzea.org/data and http://www.ricediversity.org/data/, respectively.

## Abbreviations

CW: Canopy wilting
DP: Days to pollination
ED: Ear diameter
GWAS: Genome-wide association study
LD: Linkage disequilibrium
LM: Linkage mapping
MAF: Minor allele frequency
MLM: Mixed linear model
PH: Plant height
QTLs: Quantitative trait loci
SD: Seeds per panicle
SNPs: Single nucleotide polymorphisms
