# Supplementary material for: Estimation of a significance threshold for genome-wide association studies
Source: BMC Genomics. 2019 Jul 29;20:618. doi: 10.1186/s12864-019-5992-7 (PMC6664749; doi:10.1186/s12864-019-5992-7)
Supplement: Supplementary file 1 — Figure S1. Scatter plots between significant threshold and marker-based heritability in maize, soybean, and rice. Table S1. Phenotypic data of canopy wilting (CW) and carbon isotope ratio (C13) from 346 soybean accessions previously reported by Kaler et al. (19, 20). Table S2. The R code script used for trait simulation for rice data. Similar programming can be used for other crops by changing the genotypic data. (DOCX 176 kb) [file 12864_2019_5992_MOESM1_ESM.docx]

**Figure S1.** Scatter plots between significant threshold and marker-based heritability in maize, soybean, and rice.


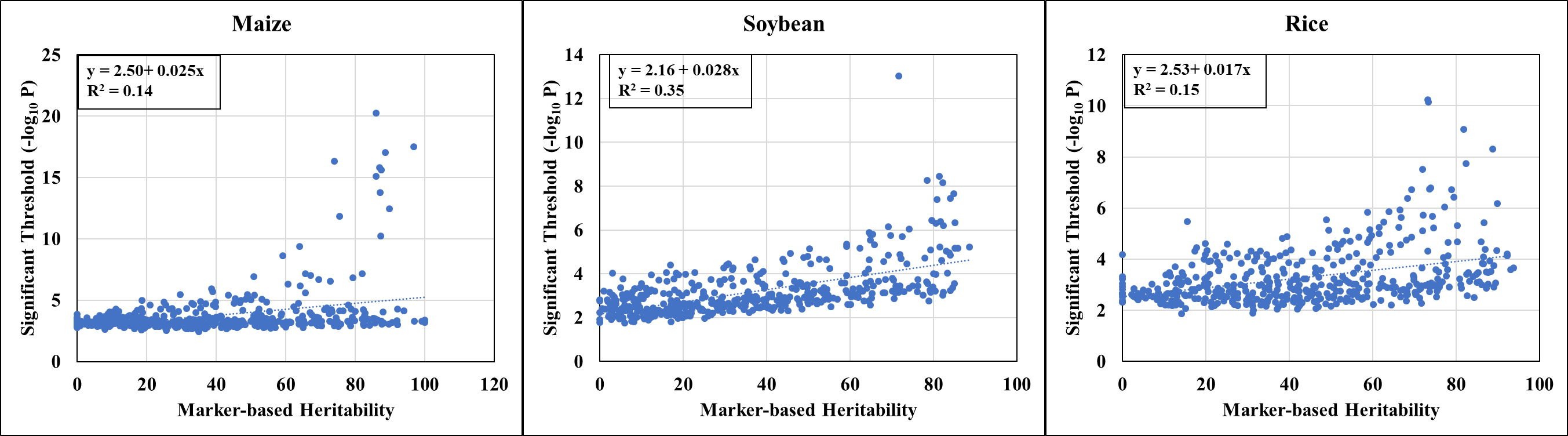


**Table S1**. Phenotypic data of canopy wilting (CW) and carbon isotope ratio (C13) from 346 soybean accessions previously reported by Kaler et al. (19, 20).

| Accession | C13 | CW |
| --- | --- | --- |
|  |  |  |
| PI424485 | -28.446 | 11.25 |
| PI507368 | -29.220 | 15.00 |
| PI398772 | -28.808 | 16.25 |
| PI398223 | -28.789 | 11.25 |
| PI398406 | -28.960 | 10.00 |
| PI416983 | -29.218 | 19.38 |
| PI417070 | -29.463 | 18.13 |
| PI424247B | -28.932 | 34.38 |
| PI507367 | -29.464 | 38.13 |
| PI509079 | -29.007 | 16.25 |
| PI594280B | -29.068 | 23.13 |
| PI594020 | -28.965 | 20.00 |
| PI602501 | -29.192 | 10.00 |
| PI603418D | -28.574 | 20.63 |
| PI404177 | -29.277 | 18.75 |
| PI567767B | -29.145 | 13.13 |
| PI603454 | -29.171 | 11.88 |
| PI603915D | -28.868 | 15.63 |
| PI424405B | -29.139 | 11.88 |
| PI597480A | -28.671 | 10.00 |
| PI592946 | -28.782 | 8.75 |
| PI424152 | -28.955 | 20.00 |
| PI592940 | -29.214 | 8.13 |
| PI603462 | -28.728 | 21.25 |
| PI398804 | -29.077 | 19.38 |
| PI424349B | -28.781 | 13.13 |
| PI423799A | -29.044 | 8.75 |
| PI398272 | -29.681 | 23.75 |
| PI424357A | -29.080 | 15.63 |
| PI424488B | -29.303 | 14.38 |
| PI424232A | -28.757 | 8.75 |
| PI567201D | -29.718 | 11.25 |
| PI398225 | -28.720 | 14.38 |
| PI424489A | -29.522 | 15.00 |
| PI424490 | -28.764 | 18.75 |
| PI424154A | -29.215 | 18.75 |
| PI423802 | -29.444 | 26.88 |
| PI432359 | -28.613 | 12.50 |
| PI398200 | -28.788 | 12.50 |
| PI398226 | -28.927 | 22.50 |
| PI424370A | -29.056 | 21.25 |
| PI424483 | -29.032 | 30.00 |
| PI567202 | -28.943 | 20.00 |
| PI408200A | -28.640 | 16.25 |
| PI398181 | -29.154 | 17.50 |
| PI424489B | -29.180 | 14.38 |
| PI561303 | -29.426 | 21.25 |
| PI592947 | -29.066 | 15.00 |
| PI398334 | -28.937 | 16.25 |
| PI398830 | -29.199 | 20.00 |
| PI417232 | -29.087 | 10.00 |
| PI458098 | -28.888 | 10.63 |
| PI567603B | -29.000 | 14.38 |
| PI398318 | -29.356 | 10.63 |
| PI398754 | -28.899 | 13.75 |
| PI408302 | -29.264 | 15.63 |
| PI423797 | -28.949 | 16.25 |
| PI424419 | -29.619 | 16.88 |
| PI424462A | -28.780 | 13.75 |
| PI567631 | -28.875 | 15.63 |
| PI398243 | -29.198 | 20.00 |
| PI458082 | -29.308 | 18.13 |
| PI603911C | -28.934 | 10.63 |
| PI398188 | -29.570 | 23.13 |
| PI567496 | -29.499 | 13.75 |
| PI408306 | -29.269 | 12.50 |
| PI398741 | -28.480 | 13.13 |
| PI417278 | -28.983 | 15.00 |
| PI424500 | -28.857 | 15.00 |
| PI567174C | -29.077 | 24.38 |
| PI398940 | -28.834 | 26.25 |
| PI398823 | -28.742 | 13.13 |
| PI408175 | -28.860 | 12.50 |
| PI273483C | -29.389 | 33.75 |
| PI398774 | -29.127 | 17.50 |
| PI424597 | -29.163 | 20.63 |
| PI603457A | -29.017 | 14.38 |
| PI398258 | -28.961 | 21.25 |
| PI567500 | -29.311 | 11.88 |
| PI404161 | -29.479 | 26.25 |
| PI398322 | -29.003 | 20.00 |
| PI398396 | -29.028 | 11.88 |
| PI408224A | -28.718 | 15.63 |
| PI424381 | -29.206 | 36.25 |
| PI597480B | -28.691 | 8.13 |
| PI594160 | -28.900 | 22.50 |
| PI408212A | -28.810 | 10.63 |
| PI398321 | -29.047 | 14.38 |
| PI408111 | -29.597 | 11.88 |
| PI408136 | -29.139 | 19.38 |
| PI408226A | -28.922 | 12.50 |
| PI407898A | -29.355 | 16.25 |
| PI424505 | -29.121 | 13.75 |
| PI424511 | -29.037 | 14.38 |
| PI424520 | -29.220 | 15.63 |
| PI567201C | -29.522 | 12.50 |
| PI404176 | -29.159 | 16.25 |
| PI424402A | -28.763 | 13.75 |
| PI398201 | -28.918 | 11.88 |
| PI424424 | -29.346 | 18.13 |
| PI423748B | -28.801 | 11.88 |
| PI398757 | -28.920 | 10.63 |
| PI408131A | -29.173 | 11.88 |
| PI424225 | -29.379 | 20.63 |
| PI597485 | -28.956 | 21.88 |
| PI398385 | -29.064 | 25.00 |
| PI398995 | -29.223 | 36.88 |
| PI424347A | -29.213 | 11.25 |
| PI603171 | -29.486 | 14.38 |
| PI360836 | -29.360 | 31.25 |
| PI408318A | -28.988 | 9.38 |
| PI417171 | -29.333 | 26.88 |
| PI603909B | -29.502 | 13.75 |
| PI398905 | -29.339 | 20.00 |
| PI398790 | -28.945 | 14.38 |
| PI408269A | -29.397 | 20.63 |
| PI567532 | -29.177 | 11.25 |
| PI408032A | -28.743 | 17.50 |
| PI603915E | -28.733 | 7.50 |
| PI398914 | -28.851 | 13.75 |
| PI407932A | -29.193 | 14.38 |
| PI408169D | -28.852 | 18.13 |
| PI408255B | -28.958 | 20.63 |
| PI424535A | -29.341 | 11.25 |
| PI398325 | -29.287 | 18.75 |
| PI423778 | -29.177 | 20.63 |
| PI424355 | -29.199 | 17.50 |
| PI398398 | -29.091 | 16.25 |
| PI398920 | -29.062 | 13.75 |
| PI417245 | -29.231 | 20.63 |
| PI424397 | -28.892 | 23.13 |
| PI567498 | -29.274 | 12.50 |
| PI398931 | -29.264 | 15.00 |
| PI408269B | -29.456 | 22.50 |
| PI423721 | -28.714 | 11.25 |
| PI424342A | -28.953 | 15.63 |
| PI424513 | -28.709 | 18.13 |
| PI438300 | -28.809 | 16.88 |
| PI398298 | -29.032 | 13.75 |
| PI424149 | -28.702 | 7.50 |
| PI458114 | -28.801 | 12.50 |
| PI398773 | -29.088 | 18.75 |
| PI399094 | -28.532 | 15.00 |
| PI408194 | -29.174 | 8.75 |
| PI361103 | -28.764 | 20.63 |
| PI398730 | -28.762 | 9.38 |
| PI398791 | -29.279 | 19.38 |
| PI398960 | -29.271 | 16.88 |
| PI399091 | -29.500 | 15.00 |
| PI424219A | -28.757 | 21.88 |
| PI438424 | -29.092 | 13.13 |
| PI398394 | -29.213 | 18.13 |
| PI399010 | -29.337 | 17.50 |
| PI408016B | -29.174 | 16.88 |
| PI567608 | -29.016 | 15.00 |
| PI408248A | -29.184 | 11.25 |
| PI423796B | -29.099 | 15.00 |
| PI424234B | -29.163 | 18.75 |
| PI424605A | -28.860 | 15.63 |
| PI424614 | -29.074 | 31.88 |
| PI458203A | -28.854 | 20.00 |
| PI424546A | -28.664 | 11.25 |
| PI603160 | -28.507 | 21.88 |
| PI603166 | -28.908 | 20.00 |
| PI442006 | -28.836 | 11.88 |
| PI398405 | -29.359 | 16.88 |
| PI424296A | -29.047 | 20.00 |
| PI424231 | -28.901 | 13.75 |
| PI274423 | -29.122 | 20.00 |
| PI424476 | -28.972 | 11.25 |
| PI424549A | -29.078 | 14.38 |
| PI398237 | -28.987 | 21.25 |
| PI398915 | -28.954 | 19.38 |
| PI398990 | -29.232 | 11.88 |
| PI398386 | -29.687 | 17.50 |
| PI398572 | -28.682 | 21.88 |
| PI398746 | -29.350 | 17.50 |
| PI399093 | -29.200 | 18.13 |
| PI398965 | -29.653 | 12.50 |
| PI578494A | -29.412 | 10.63 |
| PI399016 | -28.794 | 15.00 |
| PI408013 | -28.543 | 11.88 |
| PI424465 | -29.215 | 12.50 |
| PI399005 | -29.087 | 10.63 |
| PI417028 | -28.739 | 20.63 |
| PI398388 | -29.074 | 23.75 |
| PI507395 | -29.068 | 26.25 |
| PI424479 | -28.990 | 13.75 |
| PI408019B | -29.017 | 16.25 |
| PI424263 | -29.025 | 12.50 |
| PI408108 | -28.904 | 13.13 |
| PI532462B | -29.499 | 21.88 |
| PI398987 | -28.984 | 14.38 |
| PI407960B | -28.866 | 25.00 |
| PI603917 | -28.802 | 11.88 |
| PI407821A | -29.317 | 13.13 |
| PI408110A | -29.336 | 13.13 |
| PI408287 | -29.136 | 30.63 |
| PI507025 | -28.724 | 15.63 |
| PI398970 | -28.970 | 10.63 |
| PI424411 | -29.128 | 11.88 |
| PI597477 | -29.220 | 16.25 |
| PI398273 | -28.401 | 9.38 |
| PI398249 | -28.991 | 22.50 |
| PI398695 | -29.351 | 15.63 |
| PI404191 | -29.482 | 28.13 |
| PI417200 | -29.205 | 10.00 |
| PI603156 | -29.098 | 14.38 |
| PI398265 | -28.665 | 11.25 |
| PI398704 | -28.814 | 30.63 |
| PI398981 | -28.962 | 16.25 |
| PI416942 | -28.990 | 23.13 |
| PI398982 | -29.483 | 18.13 |
| PI424533 | -29.245 | 10.63 |
| PI574477 | -29.487 | 16.25 |
| PI398319 | -29.010 | 11.88 |
| PI408140B | -29.164 | 23.75 |
| PI424569B | -29.209 | 12.50 |
| PI407845A | -29.131 | 34.38 |
| PI399036 | -28.881 | 25.63 |
| PI407959A | -28.884 | 8.75 |
| PI424346 | -29.105 | 14.38 |
| PI398532 | -28.826 | 12.50 |
| PI423888 | -28.535 | 28.75 |
| PI398317 | -29.026 | 35.63 |
| PI417417 | -28.999 | 18.13 |
| PI438296 | -28.619 | 11.88 |
| PI567593B | -28.876 | 8.75 |
| PI404199 | -29.238 | 8.13 |
| PI360846 | -29.725 | 22.50 |
| PI417035 | -29.079 | 15.00 |
| PI603407 | -29.309 | 21.88 |
| PI442012A | -28.897 | 15.00 |
| PI404159 | -29.213 | 34.38 |
| PI407727 | -29.127 | 9.38 |
| PI424535B | -28.642 | 16.88 |
| PI399092 | -29.349 | 22.50 |
| PI398314 | -28.730 | 19.38 |
| PI407892A | -29.138 | 24.38 |
| PI398492 | -28.645 | 11.88 |
| PI592937 | -29.027 | 11.25 |
| PI407848 | -29.172 | 13.75 |
| PI408211B | -28.996 | 9.38 |
| PI603453 | -28.627 | 12.50 |
| PI417107 | -29.182 | 13.75 |
| PI404167 | -29.536 | 13.13 |
| PI603174A | -29.241 | 17.50 |
| PI424444B | -29.041 | 22.50 |
| PI407735 | -29.169 | 10.00 |
| PI538378 | -29.004 | 9.38 |
| PI597484 | -28.973 | 18.13 |
| PI416997 | -28.367 | 13.75 |
| PI424435 | -28.912 | 23.13 |
| PI399027 | -28.784 | 18.13 |
| PI567273A | -29.174 | 14.38 |
| PI567620B | -29.538 | 11.88 |
| PI407832A | -28.880 | 20.63 |
| PI567762A | -29.214 | 26.88 |
| PI398706 | -28.958 | 28.13 |
| PI407805A | -29.043 | 15.00 |
| PI567381A | -29.447 | 12.50 |
| PI408292 | -29.106 | 17.50 |
| PI507265 | -29.045 | 21.25 |
| PI430625 | -28.926 | 19.38 |
| PI561289 | -28.791 | 9.38 |
| PI567540A | -29.343 | 11.25 |
| PI424609 | -28.809 | 15.00 |
| PI603543B | -28.995 | 7.50 |
| PI603458B | -28.798 | 7.50 |
| PI424347B | -29.074 | 18.75 |
| PI408173 | -29.009 | 11.25 |
| PI423812 | -29.500 | 15.00 |
| PI424292 | -28.584 | 15.63 |
| PI377574 | -29.449 | 13.75 |
| PI399030 | -29.093 | 20.63 |
| PI423890C | -29.160 | 35.63 |
| PI507382 | -29.607 | 31.25 |
| PI406707 | -28.996 | 14.38 |
| PI567753C | -28.906 | 11.25 |
| PI567493 | -29.538 | 16.25 |
| PI567606 | -28.847 | 17.50 |
| PI427088J | -29.396 | 8.75 |
| PI398939 | -29.042 | 25.00 |
| PI567272B | -29.490 | 16.25 |
| PI399001 | -29.291 | 11.88 |
| PI407827 | -29.350 | 11.88 |
| PI430598A | -29.008 | 13.13 |
| PI458084 | -29.265 | 25.63 |
| PI417345A | -29.191 | 10.63 |
| PI398276 | -29.081 | 16.88 |
| PI567523 | -28.653 | 12.50 |
| PI398231 | -28.497 | 11.88 |
| PI438307 | -29.120 | 18.13 |
| PI507441A | -29.113 | 16.25 |
| PI507434 | -29.249 | 17.50 |
| PI424159B | -29.166 | 11.88 |
| PI507424 | -29.822 | 42.50 |
| PI603458A | -28.743 | 16.25 |
| PI407832B | -28.840 | 9.38 |
| PI360848 | -28.814 | 15.63 |
| PI407805D | -28.921 | 16.25 |
| PI398728 | -28.856 | 16.88 |
| PI567273B | -28.918 | 15.00 |
| PI567201A | -29.542 | 10.63 |
| PI603777 | -29.043 | 10.63 |
| PI597481 | -28.844 | 32.88 |
| PI398426 | -29.049 | 15.63 |
| PI594289 | -29.643 | 18.75 |
| PI408124A | -29.118 | 22.50 |
| PI423741 | -28.970 | 14.38 |
| PI398198 | -28.382 | 15.00 |
| PI398872 | -29.047 | 13.75 |
| PI594409A | -28.826 | 15.63 |
| PI532466A | -28.873 | 13.13 |
| PI567531 | -29.099 | 13.13 |
| PI507407 | -29.502 | 45.63 |
| PI424305 | -28.591 | 17.50 |
| PI442012B | -29.373 | 32.50 |
| PI424308 | -29.371 | 18.13 |
| PI398640 | -28.840 | 20.63 |
| PI594410 | -29.000 | 23.75 |
| PI507311 | -29.561 | 30.63 |
| PI408280 | -29.416 | 16.88 |
| PI567572B | -28.511 | 18.13 |
| PI398242 | -28.918 | 23.13 |
| PI567527 | -28.786 | 21.25 |
| PI567758 | -29.587 | 16.88 |
| PI532437 | -29.011 | 11.25 |
| PI538376 | -29.112 | 15.63 |
| PI398283 | -28.922 | 12.50 |
| PI507163 | -29.088 | 17.50 |
| PI424329 | -28.667 | 7.50 |
| PI508293 | -28.908 | 25.63 |
| PI424285A | -28.693 | 8.75 |
| PI398890 | -28.801 | 10.63 |
| PI424279 | -28.974 | 10.63 |
| PI398750 | -29.257 | 16.88 |
| PI506867 | -29.434 | 40.63 |
| PI538380 | -29.500 | 10.63 |
| PI597486 | -29.248 | 12.50 |
| PI423914A | -28.823 | 17.50 |
| PI442010 | -29.047 | 10.63 |
| PI438299 | -28.755 | 20.00 |
| PI423927 | -29.116 | 15.63 |
| PI567291 | -28.896 | 23.75 |
| PI612612A | -29.165 | 20.63 |

**Table S2**. The R code script used for trait simulation for rice data. Similar programming can be used for other crops by changing the genotypic data.

#rICE

D<- read.big.matrix("GNrice.txt", type="char", sep="\t", head = TRUE)

dim(D)

D=D[,2:34849]

D1=as.data.frame(as.matrix(D))

t(D1)

D2=t(D1)

QTL <- 100*(1:10) #pick 10 QTL

u <- rep(0,34848) #marker effects

u[QTL] <- 1

g <- as.vector(crossprod(D2,u))

h2 <- 0.1

y <- g + rnorm(352,mean=0,sd=sqrt((1-h2)/h2*var(g)))

write.table(y, "H10Q10.txt", sep="\t")

QTL <- 100*(1:20) #pick 20 QTL

u <- rep(0,34848) #marker effects

u[QTL] <- 1

g <- as.vector(crossprod(D2,u))

h2 <- 0.1

y <- g + rnorm(352,mean=0,sd=sqrt((1-h2)/h2*var(g)))

write.table(y, "H10Q20.txt", sep="\t")

QTL <- 100*(1:30) #pick 30 QTL

u <- rep(0,34848) #marker effects

u[QTL] <- 1

g <- as.vector(crossprod(D2,u))

h2 <- 0.1

y <- g + rnorm(352,mean=0,sd=sqrt((1-h2)/h2*var(g)))

write.table(y, "H10Q30.txt", sep="\t")

QTL <- 100*(1:40) #pick 40 QTL

u <- rep(0,34848) #marker effects

u[QTL] <- 1

g <- as.vector(crossprod(D2,u))

h2 <- 0.1

y <- g + rnorm(352,mean=0,sd=sqrt((1-h2)/h2*var(g)))

write.table(y, "H10Q40.txt", sep="\t")

QTL <- 100*(1:50) #pick 50 QTL

u <- rep(0,34848) #marker effects

u[QTL] <- 1

g <- as.vector(crossprod(D2,u))

h2 <- 0.1

y <- g + rnorm(352,mean=0,sd=sqrt((1-h2)/h2*var(g)))

write.table(y, "H10Q50.txt", sep="\t")
